# Morbidity due to acute lower respiratory infection in children with birth defects: a total population-based linked data study

**DOI:** 10.1186/1471-2431-14-80

**Published:** 2014-03-25

**Authors:** Khadra A Jama-Alol, Hannah C Moore, Peter Jacoby, Carol Bower, Deborah Lehmann

**Affiliations:** 1 School of Population Health, The University of Western Australia, Perth, Western Australia; 2 Telethon Kids Institute, The University of Western Australia, Perth, Western Australia; 3 Western Australian Register of Developmental Anomalies, Perth, Western Australia

**Keywords:** Acute lower respiratory infections, Birth defects, Aboriginal Australian children, Linked population health data, Hospitalisations

## Abstract

**Background:**

Acute lower respiratory infections (ALRIs) are leading causes of hospitalisation in children. Birth defects occur in 5% of live births in Western Australia (WA). The association between birth defects and ALRI hospitalisation is unknown.

**Methods:**

We conducted a retrospective cohort study of 245,249 singleton births in WA (1996-2005). Population-based hospitalisation data were linked to the WA Register of Developmental Anomalies to investigate ALRI hospitalisations in children with and without birth defects. We used negative binomial regression to estimate associations between birth defects and number of ALRI hospitalisations before age 2 years, adjusting for known risk factors.

**Results:**

Overall, 9% of non-Aboriginal children and 37% of Aboriginal children with birth defects had at least one ALRI admission before age 2 years. Aboriginal children (IRR 2.3, 95% CI: 1.9-2.8) and non-Aboriginal children (IRR 2.0, 95% CI: 1.8-2.2) with birth defects had higher rates of hospitalisation for an ALRI than children with no birth defects. Rates of ALRI hospitalisation varied by type of defect but were increased for all major birth defects categories, the highest rate being for children with Down syndrome (IRR 8.0, 95% CI: 5.6-11.5). The rate of ALRI hospitalisation was 3 times greater in children with multiple birth defects than in those with isolated defects.

**Conclusions:**

Children with birth defects experience higher rates of hospitalisation for ALRIs before age 2 years than children with no birth defects. Optimal vaccination coverage and immunoprophylaxis for specific categories of birth defects would assist in reducing hospitalisation rates for ALRI.

## Background

Acute lower respiratory infections (ALRIs) are a major cause of hospitalisation in young children [1]. Children with pre-existing morbidities including birth defects are at increased risk of hospitalisation for ALRI [2]. ALRIs have been reported to be the most common reason for hospitalisation in children with Down syndrome [3]. Children with birth defects such as congenital heart disease [4], Down syndrome [4,5], neuromuscular impairment [4,6] and immunodeficiency [4], have been reported to be at increased risk of respiratory syncytial virus (RSV)-associated morbidity and mortality [7,8]. However, to our knowledge, there has been no study investigating the risk of hospitalisation for all-cause ALRI associated with a comprehensive range of isolated or multiple birth defects.

ALRI hospitalisation rates are high among Indigenous populations living in industrialised countries, including Aboriginal Australians [9]. Recently, we reported that the disparity for pneumonia hospitalisations between Aboriginal and non-Aboriginal children had declined by 32-36% in Western Australia (WA) from 1996-2000 to 2001-2005 [10]. However, overall rates for ALRI remain approximately 7 times higher in Aboriginal than non-Aboriginal children in WA [10,11]. Known risk factors for ALRI hospitalisation are male gender, being born in autumn, gestational age less than 33 weeks, born by elective caesarean section, mother who has had more than 3 previous pregnancies, maternal age less than 20 years, low social economic status, residing in regional or remote areas, percent optimal birthweight <85% and asthma or smoking during pregnancy [11-15]. In WA, birth defects occur in 5% of live births, and are important causes of childhood morbidity [16,17]. However we do not know the contribution of birth defects to the overall burden of ALRI in WA.

We have previously used population-based linked data from the Western Australian Data Linkage System (WADLS) to investigate the burden and causal pathways to ALRI hospitalisation in young children [9-11]. The availability of the WA Register of Developmental Anomalies (WARDA) (formerly known as the WA Birth Defects Registry), which is a state-based registry of birth defects [18], offers a unique opportunity to investigate associations between a broad range of birth defects and hospitalisation for ALRI in the entire population of WA. In this study, population-based hospital morbidity data have been linked to WARDA data to investigate ALRI hospitalisations in children with and without birth defects. We compare the proportion of Aboriginal and non-Aboriginal children with birth defects who were hospitalised for ALRI with the proportion of children without birth defects hospitalised for ALRI. Additionally, we investigate the rate of ALRI hospitalisation in different diagnostic categories of birth defects.

## Methods

### Setting and study population

WA covers an area of 2.5 million km^2^ and in 2005 had a population of 2 million, 3% identifying as Aboriginal or Torres Strait Islander [19].

### Data extraction from the WA data linkage system

The WADLS has been operating since 1995. It constitutes a powerful source for population-based research on health outcomes, while overcoming limiting issues relating to sample size and accurate exposure and outcome ascertainment [20,21]. Linkage of administrative health data is performed by the Data Linkage Branch at the WA Department of Health using probabilistic matching and clerical review with accuracy estimated to be 99.9% [20]. These methods were used to extract information on all 245,249 singleton live births in WA between 1996 and 2005 and their associated hospitalisations for ALRI up to age 2 years and birth defects from the Midwives’ Notification System, Birth and Death Register, Hospital Morbidity Database System and WARDA. We restricted our analysis to singleton births, as multiple births may have different risk factors for ALRI than singleton births [11,22]. The study design, data extraction details and definition of Aboriginality (Aboriginal or Torres Strait Islander descent) have been described elsewhere [10,11].

### ALRI hospitalisations

ICD10 codes were introduced in July 1999. Similar to our previously published work [11], ICD9 codes for the years 1996 to June 1999 were forward mapped to ICD10 codes using mapping tables provided by the Australian National Centre for Classifications in Health. The principal diagnosis code and 20 additional diagnosis codes were used to identify ALRI admissions for the following categories: acute bronchitis (J20), acute bronchiolitis (J21), pneumonia (J12-J18, B59, B05.2, B37.1, B01.2), influenza (J10-J11), whooping cough (A37), and unspecified ALRI (J22) [11].

### Birth defects

The WARDA has population-based information on birth defects for all children born in WA since 1980 [18]. WARDA receives birth defect notifications from over 100 sources, including cytogenetics and ultrasound departments, paediatricians, and all genetic counselling clinics [17,18]. The Register definition of a birth defect is one or more structural or functional anomalies that are present at conception or occur before the end of pregnancy and are diagnosed by 6 years of age. Each birth defect (up to 10 per case) is coded according to the 5-digit British Paediatric Association ICD-9 system and defects are grouped into major diagnostic categories (nervous system defects, congenital anomalies (CA) of eye, CA of ear, face and neck, cardiovascular defects, respiratory system defects, gastrointestinal defects, uro-genital defects, musculo-skeletal defects, CA of integument, chromosome defects, and all other defects). Each birth defect is also classified as major or minor according to a method developed by the Centers for Disease Control and Prevention [17,18]. Most minor birth defects are not recorded in the Register, unless requiring treatment [17,18].

### Statistical analysis

We used negative binomial regression to calculate incidence rate ratios (IRR) with 95% confidence intervals (CI) to estimate the association between categories of birth defects (and selected individual birth defects likely to affect the respiratory system) and the number of ALRI hospitalisations during the first two years of life, adjusting for sociodemographic characteristics that we previously identified as risk factors for ALRI and were also potential confounders. These were gender, gestational age (categorised as <33 weeks, 33-34, 35-36 or ≥ 37 weeks), percent optimal birthweight [23] (categorised as <85% (low), 85-114% (normal) or ≥ 115% (high)), number of previous pregnancies, season of birth, mode of delivery, maternal age (categorised as <20 years, 20-24, 25-29, 30-34 or ≥ 35 years), maternal smoking and asthma during pregnancy, socioeconomic status using the Socioeconomic Index for Area (SEIFA codes, grouped into quantiles) and the Accessibility/Remoteness Index of Australia (ARIA) as a measure of residential remoteness (categorised as very remote, remote, outer regional, inner regional or major cities). SEIFA and ARIA scores were calculated at the collection district level, a grouping of approximately 200 dwellings and the smallest unit available for population-based analyses [11]. Information on maternal smoking during pregnancy was added to the Midwives’ Notification System in 1997 and therefore data prior to this time were excluded from the regression models [11]. Time at risk was calculated as the time period between date of birth and the end of the study period (31^st^ December 2005) or death.

Unless otherwise specified, data for Aboriginal and non-Aboriginal children were modelled separately due to the considerable difference in ALRI hospitalisation rates [11]. Chi-square comparisons between Aboriginal and non-Aboriginal children related to frequency of birth defect categories were conducted in EpiBasic (version 1.0). All other analyses were conducted using SPSS statistics software (version 19) and StataIC (version 11).

### Ethics approval

Ethics approval to conduct the study was granted by the Department of Health WA Human Research Ethics Committee (DoHWA HREC), the Western Australian Aboriginal Health and Information Ethics Committee (WAAHIEC), and the Princess Margaret Hospital for Children Ethics Committee. Access to linked data was provided by the WA Department of Health. All data files had identifying data removed before being provided to the research team.

## Results

Between 1996 and 2005 there were 13,316 (n = 5.4%) live-born singleton children with birth defects from the population of 245,249 singleton live births (4.7% in Aboriginal children, 5.5% in non-Aboriginal children). Overall, 11,977 (90%) had isolated birth defects, 1,331 (10%) had multiple birth defects and 8 cases were missing information on their birth defects category. In addition to Down syndrome, the birth defects most commonly found as one of multiple defects were musculo-skeletal defects, cardiovascular defects and CA of ear, face and neck.

Table 1 shows in order of frequency the numbers and proportions per 1000 births of Aboriginal and non-Aboriginal children by major birth defect diagnostic categories and subcategories. Uro-genital defects were the most common birth defects in non-Aboriginal children (17.2/1000 births, compared to 9.5/1000 births in Aboriginal children, p < 0.001), while musculo-skeletal defects were common among Aboriginal and non-Aboriginal children (13.7/1000 births and 13.6/1000 births, p = 0.8), as were cardiovascular defects (11.6/1000 Aboriginal births and 10.5/1000 non-Aboriginal births, p = 0.2; Table 1). Overall, 22.5% of Aboriginal children and 5.1% of non-Aboriginal children were admitted at least once for ALRI before age 2 years while 37.0% of Aboriginal children with birth defects and 9.2% of non-Aboriginal children with birth defects had at least one ALRI admission before age 2 years. Bronchiolitis accounted for 51% (n = 3178) and pneumonia for 27% (n = 1711) of ALRI hospitalisations in Aboriginal children aged less than 2 years; proportions in non-Aboriginal children were 62% (n = 8407) for bronchiolitis and 20% (n = 2724) for pneumonia. In Aboriginal children the average length of stay during an ALRI episode in those aged less than 2 years was 6.5 days in those with multiple defects, 7.4 days in those with single birth defects and 4.2 days in those with no birth defect. Equivalent figures in non-Aboriginal children were 12.1, 5.9 and 3.2 days.

**Table 1:** Frequency of birth defects in Western Australian Aboriginal and non-Aboriginal children by diagnostic category (1996-2005)

Diagnostic category^*^	**Birth defects per 1000 births (n)**	
	**Aboriginal**	**Non-Aboriginal**
Uro-genital defects	9.5 (166)	17.2 (3910)
Musculo-skeletal defects	13.7 (240)	13.6 (3088)
Diaphragmatic hernia	0.6 (11)	0.2 (56)
Cardiovascular defects	11.6 (202)	10.5 (2393)
Ventricular septal defect	6.2 (109)	6.2 (1405)
Atrial septal defect	2.2 (39)	1.7 (383)
Nervous system defects	5.3 (92)	2.4 (542)
Spina bifida	0.7 (13)	0.2 (45)
CA of eye	1.3 (23)	1.1 (260)
CA of ear, face and neck	3.6 (63)	3.1 (703)
Respiratory system defects	0.9 (15)	0.6 (135)
Gastro-intestinal defects	5.3 (92)	5.5 (1248)
Cleft palate only	1.3 (22)	1.0 (234)
Cleft lip only	0.8 (14)	0.5 (106)
Cleft lip and palate	0.9 (15)	0.5 (113)
T-OF, OA/S	0.2 (4)	0.3 (59)
CA of integument	1.7 (30)	4.8 (1091)
Chromosome defects	1.7 (30)	2.0 (450)
Down syndrome	1.0 (18)	0.9 (211)
Other	9.3 (163)	5.7 (1288)
Cystic fibrosis	0.3 (6)	0.3 (79)

Note: numbers and proportions are in order of frequency of major birth defect diagnostic categories.

^*^A child may be in more than one diagnostic category of major birth defects.

CA, Congenital Anomalies; T-OF, OA/S, Tracheo-oesophageal fistula, oesophageal atresia/stenosis.

Both Aboriginal and non-Aboriginal children with birth defects had twice the rate of hospitalisation for an ALRI before age 2 years compared with Aboriginal and non-Aboriginal children with no birth defects, after adjusting for all known risk factors (Aboriginal IRR = 2.3; 95% CI 1.9, 2.8; non-Aboriginal IRR = 2.0, 95% CI 1.8, 2.2; Table 2).

**Table 2:** Adjusted incidence rate ratio for ALRI hospitalisations among children aged <2 years according to risk factors

**Risk factor**		**Aboriginal**		**Non-Aboriginal**	
		IRR^*^	**(95% CI)**	IRR^*^	**(95% CI)**
Any birth defect		2.3	(1.9, 2.8)	2.0	(1.8, 2.2)
Gender					
	Female		Reference		Reference
	Male	1.4	(1.3, 1.5)	1.4	(1.3, 1.5)
Gestational age					
	≥37 weeks		Reference		Reference
	35-36 weeks	1.5	(1.3, 1.8)	1.7	(1.5. 1.8)
	33-34 weeks	1.7	(1.3, 2.3)	2.1	(1.7, 2.5)
	<33 weeks	3.0	(2.4, 3.9)	4.6	(4.0, 5.4)
Percent optimal birthweight					
	Normal 85-114%		Reference		Reference
	Low < 85%	1.1	(1.0, 1.2)	1.2	(1.1, 1.2)
	High ≥ 115%	1.0	(0.8, 1.2)	1.0	(0.9, 1.1)
Number of previous pregnancies					
	0		Reference		Reference
	1	1.0	(0.9, 1.2)	1.7	(1.5, 1.8)
	2	1.4	(1.1, 1.6)	2.1	(1.9, 2.3)
	≥ 3	1.7	(1.5, 2.0)	2.5	(2.4, 2.7)
Season of birth					
	Spring		Reference		Reference
	Summer	1.3	(1.1, 1.5)	1.2	(1.1, 1.2)
	Autumn	1.4	(1.2, 1.6)	1.5	(1.4, 1.6)
	Winter	1.3	(1.1, 1.4)	1.3	(1.2, 1.4)
Mode of delivery					
	Vaginal		Reference		Reference
	Instrumental	1.1	(0.9, 1.4)	1.0	(0.9, 1.0)
	Elective caesarean	1.1	(0.9, 1.3)	1.3	(1.2, 1.4)
	Emergency caesarean	1.1	(0.9, 1.3)	1.2	(1.1, 1.2)
Maternal smoking during pregnancy					
	No		Reference		Reference
	Yes	1.2	(1.1, 1.4)	1.3	(1.2, 1.4)
Maternal asthma during pregnancy					
	No		Reference		Reference
	Yes	1.1	(0.9, 1.3)	1.5	(1.4, 1.5)
Maternal age					
	≥ 35 years		Reference		Reference
	30-34 years	1.0	(0.8, 1.3)	1.2	(1.1, 1.3)
	25-29 years	1.3	(1.0, 1.6)	1.6	(1.4, 1.7)
	20-24 years	1.5	(1.2, 1.9)	2.0	(1.8, 2.2)
	< 20 years	2.0	(1.6, 2.6)	2.7	(2.4, 3.1)
SEIFA Index of disadvantage^**^					
	91-100%		Reference		Reference
	76-90%	0.9	(0.3, 2.4)	1.1	(1.0, 1.3)
	26-75%	1.5	(0.6, 3.7)	1.2	(1.0, 1.3)
	11-25%	1.5	(0.6, 3.8)	1.3	(1.2, 1.5)
	0-10%	1.8	(0.7, 4.3)	1.4	(1.2, 1.6)
Accessibility/Remoteness index of Australia					
	Very remote		Reference		Reference
	Remote	0.6	(0.5, 0.7)	1.5	(1.1, 1.9)
	Outer regional	0.7	(0.6, 0.8)	1.7	(1.3, 2.2)
	Inner regional	0.5	(0.4, 0.6)	1.1	(0.8, 1.5)
	Major cities	0.5	(0.4, 0.6)	1.2	(0.9, 1.6)

*IRR, incidence rate ratio.

^**^91-100% is least disadvantaged and 0-10% is most disadvantaged.

To investigate any differences between types of ALRI, we investigated the association between birth defects and bronchiolitis and pneumonia. As the vast majority of bronchiolitis hospitalisations occur in the first year of life, the outcome for bronchiolitis was restricted to that age group. The rates of admission for any ALRI before age 2 years, bronchiolitis in the first year of life, or pneumonia before age 2 years were higher in Aboriginal and non-Aboriginal children with multiple birth defects than in those with isolated birth defects (Table 3). In particular, after adjusting for other known risk factors, there was a strong positive association between multiple birth defects and hospitalisation for pneumonia in the first 2 years of life in both Aboriginal (IRR = 5.0, 95% CI: 2.9, 8.6) and non-Aboriginal children (IRR = 6.0, 95% CI: 4.4, 8.2; Table 3).

**Table 3:** Adjusted incidence rate ratio for ALRI hospitalisations in children with isolated or multiple birth defects (BDs)

**Hospitalisation diagnosis**	**Aboriginal**		**Non- Aboriginal**	
	**Isolated BDs**	**Multiple BDs**	**Isolated BDs**	**Multiple BDs**
	IRR* **(95%CI)**	IRR* **(95%CI)**	IRR* **(95%CI)**	IRR* **(95%CI)**
Any ALRI <24 months	2.0 (1.6, 2.5)	3.4 (2.3, 5.0)	1.8 (1.6, 1.9)	4.4 (3.6, 5.4)
Bronchiolitis <12 months	2.0 (1.5, 2.7)	1.7 (1.0, 3.0)	1.5 (1.4, 1.7)	3.4 (2.6, 4.4)
Pneumonia <24 months	1.5 (1.0, 2.2)	5.0 (2.9, 8.6)	1.8 (1.5, 2.1)	6.0 (4.4, 8.2)

*IRR, incidence rate ratio.

For each model, the reference group is children with no birth defects.

Adjusted for gender, gestational age, season of birth, mode of delivery, number previous pregnancies, per cent optimal birthweight (POBW), maternal age, maternal asthma during pregnancy, maternal smoking during pregnancy, and socioeconomic indices for areas accessibility/remoteness index of Australia [11].

Due to small numbers of individual birth defects in the major categories and subcategories, we combined data from Aboriginal and non-Aboriginal children to investigate the association between different types of birth defects and ALRI hospitalisations. After adjusting for all known risk factors, there was a positive association between all major categories of birth defects and hospitalisation for any ALRI in the first 2 years of life, bronchiolitis in the first year of life and pneumonia in the first 2 years of life (Table 4). There were particularly high hospitalisation rates for pneumonia associated with Down syndrome (IRR = 14) and with tracheo-oesophageal fistula, oesophageal atresia/stenosis (IRR = 16), although the latter was based on only 4 cases (Table 4). Separate analyses of specific diagnoses of ALRI other than pneumonia or bronchiolitis was not possible due to limited numbers of cases.

**Table 4:** Adjusted incidence rate ratio for ALRI hospitalisations in Aboriginal and non-Aboriginal children according to birth defect diagnostic category

**Diagnostic category**	**Any ALRI**		**Bronchiolitis**		**Pneumonia**	
	**<24 months**		**<12 months**		**<24 months**	
	IRR*	**(95% CI)**	IRR*	**(95% CI)**	IRR*	**(95% CI)**
Uro-genital defects	1.6	(1.3, 1.8)	1.4	(1.1, 1.6)	1.4	(1.0, 1.8)
Musculo-skeletal defects	1.8	(1.5, 2.1)	1.6	(1.3, 1.9)	1.9	(1.4, 2.5)
Diaphragmatic hernia	2.4	(0.9, 6.4)	1.5	(0.3, 7.5)	4.3	(1.1, 16.7)
Cardiovascular defects	3.1	(2.7, 3.6)	2.5	(2.0, 3.0)	3.3	(2.6, 4.2)
Ventricular septal defect	2.6	(2.2, 3.2)	2.2	(1.6, 2.8)	2.7	(1.9, 3.8)
Atrial septal defect	4.7	(3.4, 6.3)	2.8	(1.7, 4.3)	6.9	(4.4, 10.8)
Nervous system defects	3.3	(2.5, 4.3)	2.4	(1.6, 3.5)	4.4	(2.8, 6.7)
Spina bifida	2.9	(0.9, 9.4)	4.3	(1.1, 15.9)	-	-**
CA of eye	3.4	(2.2, 5.2)	1.9	(1.0, 3.6)	4.9	(2.5, 9.3)
CA of ear, face and neck	4.5	(3.6, 5.6)	3.9	(2.9, 6.3)	4.2	(2.8, 6.4)
Respiratory system defects	2.9	(1.6, 5.0)	1.8	(0.8, 4.0)	3.6	(1.5, 8.9)
Gastro-intestinal defects	2.2	(1.8, 2.7)	1.9	(1.4, 2.6)	2.6	(1.7, 3.8)
Cleft palate only	2.2	(1.3, 3.6)	2.2	(1.1, 4.2)	1.7	(0.6, 4.8)
Cleft lip only	0.5	(0.1, 1.6)	-	-**	1.4	(0.3, 6.0)
Cleft lip and palate	2.5	(1.2, 4.8)	4.0	(1.9, 8.6)	0.9	(0.1, 6.9)
T-OF, OA/S	7.4	(3.1, 17.7)	3.5	(0.9, 13.1)	15.8	(5.1, 48.6)
CA of integument	1.6	(1.2, 2.1)	1.9	(1.3, 2.6)	1.1	(0.6, 2.0)
Chromosome defects	6.7	(5.1, 8.7)	4.7	(3.2, 6.8)	9.9	(6.7, 14.4)
Down syndrome	8.0	(5.6, 11.5)	5.4	(3.3, 9.0)	13.6	(8.4, 21.9)

*IRR, incidence rate ratio.

**too few cases for analysis.

For each model, the reference group is children with no birth defects.

Adjusted for gender, gestational age, season of birth, mode of delivery, number previous pregnancies, per cent optimal birthweight (POBW), maternal age, maternal asthma during pregnancy, maternal smoking during pregnancy, and socioeconomic indices for areas accessibility/remoteness index of Australia [11].

CA, Congenital Anomalies; T-OF, OA/S, Tracheo-oesophageal fistula, oesophageal atresia/stenosis.

## Discussion

Using total population-based data, we have shown that children in WA with any birth defect have a two-fold higher rate of hospitalisation for ALRI in the first 2 years of life than children with no birth defects. In addition, the rates of hospitalisation for ALRI were higher in children with multiple birth defects than children with isolated birth defects. The rates were increased in all major birth defect categories and for both bronchiolitis in the first year of life and pneumonia before the age of two years. Our findings are consistent with previously reported studies that have generally investigated a pathogen-specific ALRI such as that caused by RSV or have only considered a specific birth defect or syndrome such as Down syndrome [3,7]. To our knowledge, these population-based data are the first contemporary findings to report the rate of hospitalisation for ALRI in both Aboriginal and non-Aboriginal children with and without birth defects. Unlike other risk factors we have reported previously [11], our results here show remarkable similarities in the rate of hospitalisation for ALRI with birth defects in both Aboriginal and non-Aboriginal children.

There are a number of reasons why children with birth defects are at increased rate of hospitalisation for ALRI which will vary according to the type of birth defect. There may be anatomical defects that increase risk of pathogens entering the upper respiratory tract and lung, they may be born preterm, of low birthweight and children may have a compromised immune system making them more susceptible to infection. Children with Down syndrome may have abnormal airway anatomy, congenital heart disease and hypotonia impairing swallowing and putting them at risk of aspiration. Surgery under anaesthetic increases risk of ALRI and many children with birth defects require surgical management. For all these reasons, unless specifically contraindicated, it is important that children with birth defects receive vaccines according to standard schedules, specifically pneumococcal, *Haemophilus influenzae* type b, pertussis, measles and influenza vaccines for prevention of ALRIs.

There are several limitations to our study. Firstly, our linked hospitalisation data only included hospitalisations with a respiratory infection diagnosis and not all admissions for children with birth defects. Therefore, we do not know whether the birth defects were surgically corrected and, if so, whether this occurred prior to any hospitalisations with a diagnosis of ALRI. Secondly, we do not have information on other potential risk factors such as breast feeding and duration of breast feeding, the child’s immunisation status and child care attendance. Lastly, we were unable to determine if and where individuals moved within or outside the state during the study period. Therefore information related to socioeconomic status and the accessibility/remoteness index may have changed between the time of birth and time of hospitalisation. However we believe this is likely to have had little impact on our results [24] as, generally, our results highlight information on maternal and infant factors in the antenatal and natal period and analysis was restricted to hospitalisations in the first 2 years of life and we would not expect individuals to move very far from their place of birth in their first 2 years of life. Despite these limitations our study is total population-based and provides important information that can assist in planning effective health care strategies based on individual category and subcategories of birth defects and for prevention of hospitalisations for ALRI among children with birth defects. We would now encourage other research groups with access to administrative health care datasets similar to these to replicate the analyses we have conducted here which will provide further evidence to assist in planning future health care strategies for children with birth defects.

Immunoprophylaxis with the RSV monoclonal antibody, palivizumab, is effective in reducing severe RSV-related hospitalisations, mostly associated with bronchiolitis and pneumonia [25]. Palivizumab has also been found to reduce the risk of hospitalisations in babies with congenital heart disease and monthly immunoprophylaxis is now recommended for infants with congenital heart disease or chronic lung disease [25]. Our results provide further evidence to support such recommendations since children with congenital heart defects were more than twice as likely to be hospitalised for bronchiolitis in the first year of life and for pneumonia in the first 2 years of their life than children with no birth defects. In WA during the period of our study palivizumab was prescribed on a case-by-case basis, but since 2009 has been recommended during the RSV season for children with severe cardiac conditions and it is currently being considered for those with chronic respiratory conditions as well. While the cost of palivizumab is high, in view of the increased risk of hospitalisation for bronchiolitis and pneumonia in children with birth defects, consideration should be given to offering palivizumab prophylaxis to children with serious birth defects, particularly those with multiple defects. From our findings we suggest randomised controlled trials should be conducted to determine whether immunoprophylaxis for children with nervous system defects, congenital anomalies of ear, face and neck, trachea-oesophageal fistulae or oesophageal atresia or stenosis, cleft lip and palate, diaphragmatic hernia and any chromosomal defect is effective in reducing hospitalisations for bronchiolitis or pneumonia. Any such trial is likely to be difficult due to the large sample size that will be needed to adequately detect a significant difference. A further consideration is that prophylaxis should be restricted to the months with high rates of RSV infection to reduce cost, and timing will vary according to geographic location and climate [26].

## Conclusions

In summary this total population-based study has provided useful information on the rate of hospitalisation for ALRI for a broad range of birth defects in Aboriginal and non-Aboriginal Australian children. Primary prevention of and prenatal screening for birth defects, optimal vaccination coverage and immunoprophylaxis for specific categories of birth defects will assist in reducing hospitalisation rates for ALRI.

## Competing interests

The authors declare that they have no competing interests.

## Authors’ contributions

All authors developed the study design. KhJA cleaned and analysed the birth defect data and drafted the manuscript. HM cleaned the original datasets from the previous analysis, conducted part of the current data analysis and assisted in the preparation of the manuscript. PJ provided expert statistical advice. CB provided expert clinical advice on the birth defects data and interpretation of results. DL provided expert advice on all issues and provided support throughout the study and critically reviewed the draft manuscript. All authors read and approved the final manuscript prior to submission.

## Pre-publication history

The pre-publication history for this paper can be accessed here:

http://www.biomedcentral.com/1471-2431/14/80/prepub
